# The genetic diversity of *Strongyloides papillosus* in Pakistani goats revealed by whole genome sequencing

**DOI:** 10.1186/s13071-024-06626-6

**Published:** 2024-12-20

**Authors:** Kiran Afshan, Yuchen Liu, Mark Viney

**Affiliations:** 1 Department of Zoology, Faculty of Biological Sciences, Quaid-i-Azam University, Islamabad, 45320 Pakistan; 2 Department of Evolution, Ecology and Behaviour, University of Liverpool, Liverpool, L69 7ZB UK

**Keywords:** Strongyloides, *Strongyloides papillosus*, Population genomics

## Abstract

**Background:**

*Strongyloides* nematodes are livestock parasites, and *Strongyloides papillosus* infecting ruminant livestock can cause disease. Recent genomic analysis of several *Strongyloides* species is now facilitating population genomic analyses of natural *Strongyloides* infections, for example finding that *Strongyloides ratti* in wild UK rats exists as an assemblage of long-lived, asexual lineages.

**Methods:**

Here we have initiated an investigation into the population genomics of *S. papillosus* in goats in Pakistan. We sampled *Strongyloides* from goat faeces and then whole genome sequenced individual larvae.

**Results:**

We find that *S. papillosus* is common, with a prevalence of 28%; that the population is genetically diverse and that individual goats commonly have mixed-genotype infections, and that there is evidence of admixture in only ca. 20% of worms.

**Conclusions:**

These results now provoke further questions about the host range of different *S. papillosus* genotypes that can be investigated by further population genomic analyses in the future.

**Graphical Abstract:**

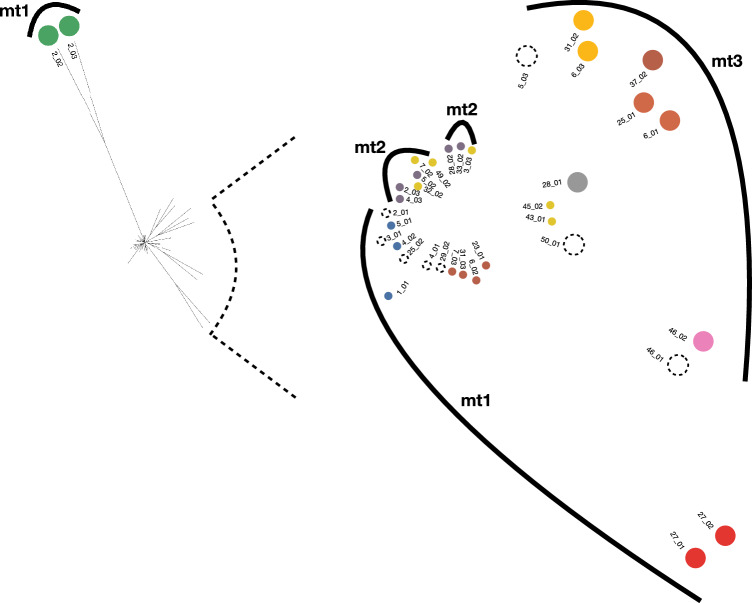

**Supplementary Information:**

The online version contains supplementary material available at 10.1186/s13071-024-06626-6.

## Background

*Strongyloides* is a genus of gut parasitic nematodes that infects a wide variety of terrestrial vertebrates. *Strongyloides papillosus* has been reported to parasitise sheep, goats, and cattle and can be experimentally maintained in rabbits [1]. However, the species identity of *Strongyloides* infecting these host species has been questioned. Sequence analysis of the rRNA coding gene of *Strongyloides* from cattle and sheep found two different forms that appeared to be substantially (though not completely) host specific [2]. The names *S. papillosus* and *Strongyloides vituli* for parasites from sheep and cattle, respectively, were originally suggested by [3] (though not widely used) and reinitiated by [2], with further support for *S. vituli* infection in cattle [4]. More generally, the host specificity of *Strongyloides* species remains unexplored [5]. Most *Strongyloides* species have been described morphologically, often from only some stages of the life cycle, which makes it difficult to define not only a *Strongyloides* species but also its host range. However, population genetic analyses of *Strongyloides* has the potential to resolve such questions.

The parasitic phase of *S. papillosus* (in common with other *Strongyloides species*) consists only of adult female worms that live in the mucosal epithelium of the small intestine and reproduce by mitotic parthenogenesis. Eggs are shed in the host faeces, ultimately resulting in infective, filariform third-stage larvae (iL3s). These iL3s can develop either, directly, via two larval moults, or, indirectly, as the progeny of a facultative adult dioecious free-living generation that reproduces sexually [6]. Hosts become infected by percutaneous infection by iL3s [7]. The parthenogenetic reproduction of the parasitic female worms means that the larvae passed in faeces are genetic copies of their mother that is resident in the host gut. The only opportunity for sexual reproduction is when free-living male and female worms are present (presumably in faeces or nearby soil). The factors that control the occurrence of this free-living adult generation are not fully understood, but in *S. ratti* in rats this varies among *S. ratti* genotypes and is affected by both the temperature external to the host and the host immune response [8]. From a population genetic perspective, the facultative nature of sexual reproduction may mean that the population genetic structure of *Strongyloides* deviates from that predicted by Hardy-Weinberg expectations.

*Strongyloides papillosus* can be a common infection of ruminants, with a prevalence of up to 58% reported in lambs, though the prevalence differs widely geographically and among different livestock management systems [9]. *Strongyloides papillosus* can cause diarrhoea, dehydration, anorexia, anaemia, and malnutrition, especially in young animals [9]. High intensity infections can cause serious strongyloidiasis, which can be fatal [1]. Juvenile animals are most susceptible to heavy infection upon first exposure, but rapidly develop acquired immunity to future *S papillosus* infections [7]. *Strongyloides papillosus* can be treated with anthelminthic drugs such as macrocyclic lactones (e.g. ivermectin) and albendazole [9]. Rabbits can also be infected with *S. papillosus* and as such can be used as an experimental model for *S. papillosus* [9].

The genome of *S. papillosus* has been sequenced, together with three other *Strongyloides* species [10]. The *S. papillosus* genome is estimated to be 60 Mbp, though it is less well assembled than the other *Strongyloides* species such that the estimate of its genome size may not be very accurate. Nonetheless, the *S. papillosus* genome assembly can be used for population genomic analyses of this species.

Population genetic analyses of parasitic nematodes of livestock have shown that there is very high gene flow and an absence of parasite population structure [11]. This was thought to be due to both the often very large effective population sizes of the nematodes and the extensive host movement that occurs as part of commercial farming. For nematode parasites of wildlife species, diversity of the observed parasite population structures was observed, with this dependant on the specific host and parasite biology [12]. There have been previous population genomic analyses of *Strongyloides*. Specifically, *S. ratti* infecting wild rats in the UK was found to exist as a mixture of mainly asexual lineage parasites that were widely dispersed across the host population, findings that were consistent with those of earlier, UK-wide studies [13, 14].

Here we report an initial population genomic analysis of *S. papillosus* in goats in Pakistan where we used single-worm, whole-genome sequencing. We describe the patterns of genetic diversity among these worms and find evidence of mixed-genotype infections in hosts, but limited admixture.

## Methods

### Study area, faecal sampling and culturing

This study was approved by the bioethical committee of Quaid-i-Azam University, Islamabad, Pakistan. Sampling was conducted at four slaughterhouses in Rawalpindi City, with the slaughterhouses having a minimum of 9 to a maximum of 32 km between them (Supplementary Fig. 1). Faecal samples were collected directly from the rectums of 150 slaughtered goats using sterile disposable plastic gloves, with fresh gloves used for each goat to avoid cross-contamination. The faeces were cultured by mixing a few grammes of fresh faeces with an approximately equal volume of wet charcoal, which was then kept at room temperature for up to 13 days when individual iL3s were collected (Supplementary Table 1); these were identified as *Strongyloides* by morphological examination [15]; no free-living adult males or females were observed.

### DNA extraction and genome sequencing of single larvae

The DNA extraction protocol is based on the method of Cole et al., 2023, but adapted for ethanol-stored worms according to [15]. Pools of larvae from each host were processed separately, individual larvae in TE were placed into wells of a 96-well plate; lysis buffer was added to give a final concentration of 200 mM NaCl, 100 mM Tris–HCl (pH 8.5), 50 mM EDTA (pH8), 0.5% w/v SDS, 0.9 mg/ml Proteinase K, and 45 mM dithiothreitol. Plates were then sealed and held at 60 °C for 2 h with gentle agitation, after which they were held at 85 °C for 15 min to inactivate the Proteinase K. Lysates were then stored at − 80 °C before whole genome sequencing.

Whole genome sequencing was performed by the Centre for Genomic Research, University of Liverpool. Illumina libraries were prepared using the NEB FS Ultra 2 DNA kit at half of the manufacturer’s specified reaction volumes.

### Sequence analyses

We whole genome sequenced 114 *S. papillosus* iL3s, mapped the sequence reads using Bowtie2 to the *S. papillosus* reference genome (PRJEB525, WBPS18 available from WormBase-ParasiteSite; parasite.wormbase.org), and calculated the average depth and coverage across the genome of each alignment using SAMtools. Of these 114 larvae, 37 passed the quality control test by having a genome coverage of at least 70% and an average depth of at least 10, and these samples were then analysed further (Supplementary Table 1).

Single nucleotide polymorphism (SNP) calling was performed using SAMtools. We used VCFtools to filter SNPs using the criteria that (i) they had a mean mapping quality of at least 20, (ii) they had a QUAL score of at least 20, (iii) SNP loci had a minor allele frequency of ≥ 0.02, and (iv) their mean depth was between the 10 and 90% percentiles of the whole data set, which was 6 and 21, respectively. Among the 37 *S. papillosus* genome sequences, nucleotides that were identical among all samples (but different from the PRJEB525 reference genome) were removed.

Using the filtered SNP set of the 37 *S. papillosus* iL3s, we performed population structure analysis by calculating the pairwise genetic similarity among the 37 worms and then (i) using principal component analysis (PCA) in PLINK 1.9 and Tassel v.5 with plots visualized in R studio v.4.2.1; (ii) constructing a neighbour-joining tree in Tassel, visualising the tree in iTOL; and (iii) using ADMIXTURE version 1.3.0 [16] analysis, with linkage disequilibrium (LD)-based pruning, which was done with window sizes of 50 kb, step sizes of 10 SNPs, and a *r*^2^ threshold of 0.1. ADMIXTURE was run for *k* = 2–15 and results plotted in R studio v.4.2.1. To investigate the genetic distance among worms we calculated the identity by distance (IBD) on the filtered SNP set in PLINK. IBD values were categorised into two pairwise comparison groups: (i) from within the same host and (ii) among different hosts.

The *S. papillosus* reference mitochondrial genome was obtained from NCBI GenBank, NC_028622.1. We generated maximum likelihood trees for the mitochondrial genomes of the 37 larvae, producing consensus fasta sequences for each; where an individual was heterozygous the reference allele was applied. Sequences were aligned with MAFFT [17] using the FF-NST-1 fast alignment method. Maximum likelihood tree estimation was carried out utilizing RaxML [18], applying a general time reversible gamma model of substitution rate heterogeneity and rapid bootstrapping with 100 replicates and visualized in ITOL.

It has been suggested that there are two species of *Strongyloides* that infect ruminants, *S. papillosus* and *S. vituli*, which can be differentiated by their rRNA sequence [2, 4], and a complete mitochondrial genome sequence has also been reported for *S. vituli* [19]. For the 37 samples, we analysed the rRNA and mitochondrial sequences to confirm their species identity.

## Results

We sampled 150 goats of which 42 (28%) were faecal culture positive for *Strongyloides*. In all we obtained 526 iL3s, ranging from 7–15 iL3s per infected goat. We sequenced 114 individual iL3s of which 37 generated sufficiently good data and were taken forward for further analysis. These 37 larvae were obtained from 21 goats. For these 37 this resulted in sequence data with an average genome coverage of 89% (range 74–94%) and an average read depth of 21 (range 9.7–69), giving an average genome coverage size of 53.6 Mb. We detected 11,023 filtered SNPs in the nuclear genome, giving an average SNP density of 0. 2 SNPs per 10 kb. This alignment of reads to the *S. papillosus* genome is consistent with identifying these larvae as *S. papillosus* rather than *S. vituli*. We also analysed the rRNA and mitochondrial genome sequence of the 37 samples, which confirmed their identity as *S. papillosus*.

**Fig. 1:**
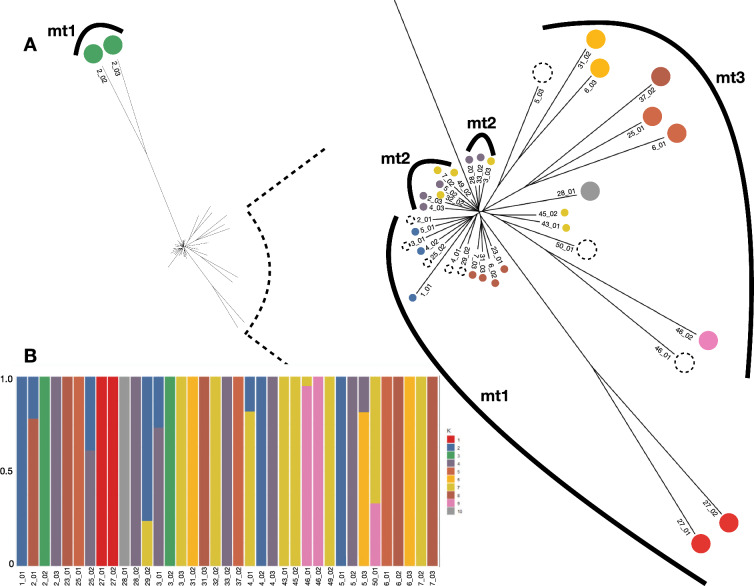
Population genomic analyses of 37 *Strongyloides* *papillosus* larvae from 21 goat hosts. **A** Neighbour-joining tree of the nuclear genome (full view to the left and the central region enlarged to the right), with individual worms coloured according to the ADMIXTURE groups, with the eight admixtured worms shown as dotted circles and the mitochondrial clades shown as numbered (mt1, mt2, mt3) arcs and **B** ADMIXTURE analysis showing the degree of ancestry on the y-axis, with k = 10. In (**A**), the two worms from ADMIXTURE group 3 (green) are from different hosts; two worms in ADMIXTURE group 1 (red) are from the same host. Larval samples are identified as host number_larva number; these numbers correspond to those in Supplementary Fig. 2, where the identification numbers are preceded by ‘pk’

To investigate the genetic relationship among the 37 iL3s we calculated the pairwise nuclear genetic similarity among the larvae and used this to construct a neighbour joining tree (Fig. 1A). The shape of this tree showed that there was extensive genetic heterogeneity among the worms. In this tree most (33 worms) were on short-length branches, showing their relatively close genetic similarity. However, two pairs of worms (two of which were from a single host, the other two from two different hosts) were on long, distant branches in the tree, showing their relative genetic dissimilarity from the other 33 worms. A PCA of the same data also showed three clusters of iL3s, with 33 iL3s in a single cluster, and then two smaller clusters each consisting of the same two pairs of divergent iL3s (Supplementary Fig. 2). We investigated the data for evidence of admixture, with the results for k = 10 [cross validation (CV) = 0.13] showing that only eight worms (22%) had evidence of admixture (Fig. 1B). Examining admixture results for other values of k also showed low levels of admixture specifically for k = 3, 7, and 15 and then 6, 3, and 6 worms showed admixture, respectively (Supplementary Fig. 3).

The mitotic parthenogenetic reproduction of *Strongyloides* parasitic females means that offspring of a single parasitic female worm will be genetically identical, so that genetically identical iL3s would be observed in host faeces. Conversely, if hosts are infected with genetically different parasitic females, then genetically diverse iL3s will be present in host faeces. With this in mind, we considered hosts from whom multiple iL3s were sequenced (Supplementary Table 1). Among the 21 hosts, 11 each had more than 1 worm sequenced, giving a total of 27 worms (5 hosts with 3 worms; 6 hosts with 2 worms). We compared the average genetic distance of worms from within hosts with worms from among different hosts, finding that there was little difference: mean (SD) within hosts 0.063 (0.068) but 0.059 (0.059) among hosts. This suggests that hosts may harbour genetically different worms. We asked whether worms from a single host were in one or more k = 10 ADMIXTURE-defined groups and/or were admixtured. We found that 10 of the 11 hosts contained worms from more than one ADMIXTURE-defined group and/or were admixtured. This suggests that multi-genotype *S. papillosus* infections are common in goats in Pakistan.

**Fig. 2:**
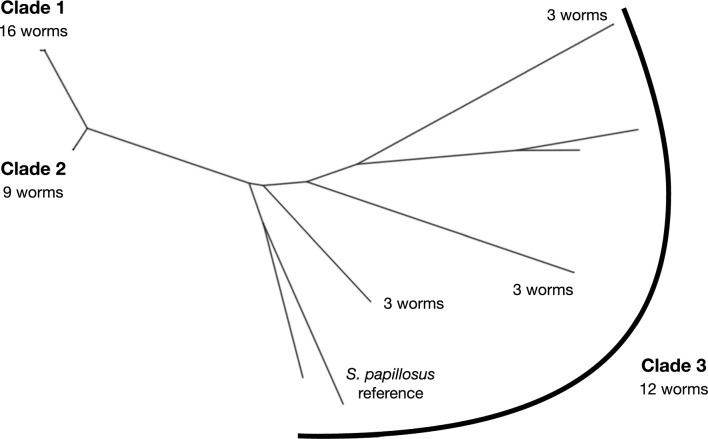
A maximum likelihood tree of the mitochondrial genome of the 37 samples and the *Strongyloides* *papillosus* reference mitochondrial genome

For the mitochondrial genome, we detected 357 SNPs and used these to construct a maximum likelihood tree of the 37 iL3 and the reference *S. papillosus* mitochondrial genome. This showed that there were three *S. papillosus* clades (Fig. 2). Two clades contained the most (16 and 9 iL3s) iL3s, with the third clade containing 12 iL3s that were more divergent. This third clade also contained the *S. papillosus* reference mitochondrial genome (Fig. 2).

As for the nuclear genome, we considered the 11 hosts that each had more than one sequenced worm. We found that nine hosts contained worms that were in more than one mitochondrial clade. This is further evidence of mixed *S. papillosus* genotype infections in these hosts. Interestingly, the four iL3s with divergent nuclear genomes (Fig. 1A) were within mitochondrial clade 1 (Fig. 2). This result therefore shows some divergence in the genetic relationship among iL3s as judged by nuclear and mitochondrial data.

We compared the nuclear and mitochondrial trees. This showed that, with the exception of one nuclear clade, the three mitochondrial clades grouped related nuclear clades (Fig. 1A). The exception is the highly divergent nuclear clade, which belongs to mitochondrial clade 1, whereas its closest neighbours belong to mitochondrial clade 2.

## Discussion

This work has used whole genome sequencing to describe the genetic diversity of *S. papillosus* in goats in Pakistan. We found that *S. papillosus* is a common infection of goats in Pakistan, with a prevalence of 28%. *Strongyloides papillosus* can infect a number of ruminant species, and its prevalence in these other species remains to be discovered. Given that *S. papillosus* is able to infect a number of different potential host species, there is the possibility for among-host species transmission of *S. papillosus*. Such sharing of parasites among different host species has the potential to genetically structure the parasite population, with this possibility being dependant on the amount of within, versus between, host species transmission.

This work revealed evidence of extensive genetic diversity among the parasites sampled for both the nuclear and mitochondrial genomes. The mitotic parthenogenetic reproduction of *Strongyloides* parasitic females means that their offspring will be genetically identical, though sequencing errors will not result in identical gene sequences. Our data show that mixed-genotype infections are common in the sampled goats, which is likely due to goats being repeatedly exposed to infection from a genetically diverse *S. papillosus* population. This pattern is broadly consistent with patterns of population genetics seen in other parasitic nematodes [11]. Among the parasites sampled, a minority were genetically relatively more divergent. Specifically, by analysis of nuclear genome, 4 of the 37 worms were more distinct. This might point to the existence of even higher levels of genetic diversity in the *S. papillosus* population, which might be revealed by more extensive sampling in goats. Furthermore, because *S. papillosus* can infect different ruminant species, the more divergent genotypes we observed may be because these are parasites whose host preference (but not restriction) is for species other than goats. However, further sampling and genetic analysis are needed to understand the distribution of *S. papillosus* among different ruminant host species and the relationship of *S. papillosus* and *S. vituli* [2]. The results of the mitochondrial genome analysis are broadly consistent with the nuclear genome analysis, showing three distinct clades with the third clade containing mitochondrially diverse worms (and the *S. papillosus* reference genome). Again, these results may potentially indicate a diversity of parasites that infect a range of host species, of which only a small portion has been sampled in the current study. However, it is notable that the grouping of parasites by their nuclear and mitochondrial genomes is not fully concordant. This is indicative of different histories of these two genomes in some of the parasites that we have sampled, which has also been observed in *Ascaris* [20].

The *Strongyloides* life cycle is obligatorily parthenogenetic with facultative sexual reproduction, where the degree of sexual reproduction varies among species, geographical location, and genotype [8]. The amount of sexual reproduction in *S. papillosus* that we have sampled is unknown, but during the culture of faeces we did not observe any free-living adult stages, suggestive of rare or no sexual reproduction. A previous analysis of wild *S. ratti* in the UK found that it consisted of a mixture of long-lived asexual lineages [14], and a similar pattern may exist among the *S. papillosus* that we sampled, although further study is required to confirm this.

To extend and develop this work and to better understand the population genomics of *S. papillosus* it would be desirable to more extensively sample larvae from a diversity of sympatric host ruminants. This will allow an analysis of the degree of partitioning, if any, of *S. papillosus* genetic diversity among different host species. Future work would also be facilitated by a better, more complete assembly of the *S. papillosus* genome, which could be achieved by long-read DNA sequencing. Analysis of diverse, wild genotypes of *S. ratti* has discovered clusters of parasitism genes [10] that are hyperdiverse compared with the rest of the *S. ratti* genome [12]. It would be interesting to study this in *S. papillosus* too, but this will also require a more complete genome assembly.

## Supplementary Information

Supplementary Material 1. Figure 1. Map showing the location of the slaughterhouses.

Supplementary Material 2. Figure 2. Principal component analysis showing the first two principal components, which accounts for 38% of the variance. Larval sample are identified as host number_larva number, preceded by ‘pk’. These numbers correspond to those in Figure 1 where the preceding ‘pk’ is omitted.

Supplementary Material 3. Figure 3. ADMIXTURE analyses for k = 3, 7, and 15.

Supplementary Material 4. Table.

## Data Availability

No datasets were generated or analysed during the current study.

## Abbreviations

CV: Cross validation
EDTA: Ethylenediaminetetraacetic acid
IBD: Identity by distance
LD: Linkage disequilibrium
NEB: New England Biolabs
PCA: Principal component analysis
SD: Standard deviation
SDS: Sodium dodecyl sulphate
SNP: Single nucleotide polymorphism
