# Supplementary figures and images for: The genetic diversity of Strongyloides papillosus in Pakistani goats revealed by whole genome sequencing
Source: Parasit Vectors. 2024 Dec 20;17:527. doi: 10.1186/s13071-024-06626-6 (PMC11662772; doi:10.1186/s13071-024-06626-6)

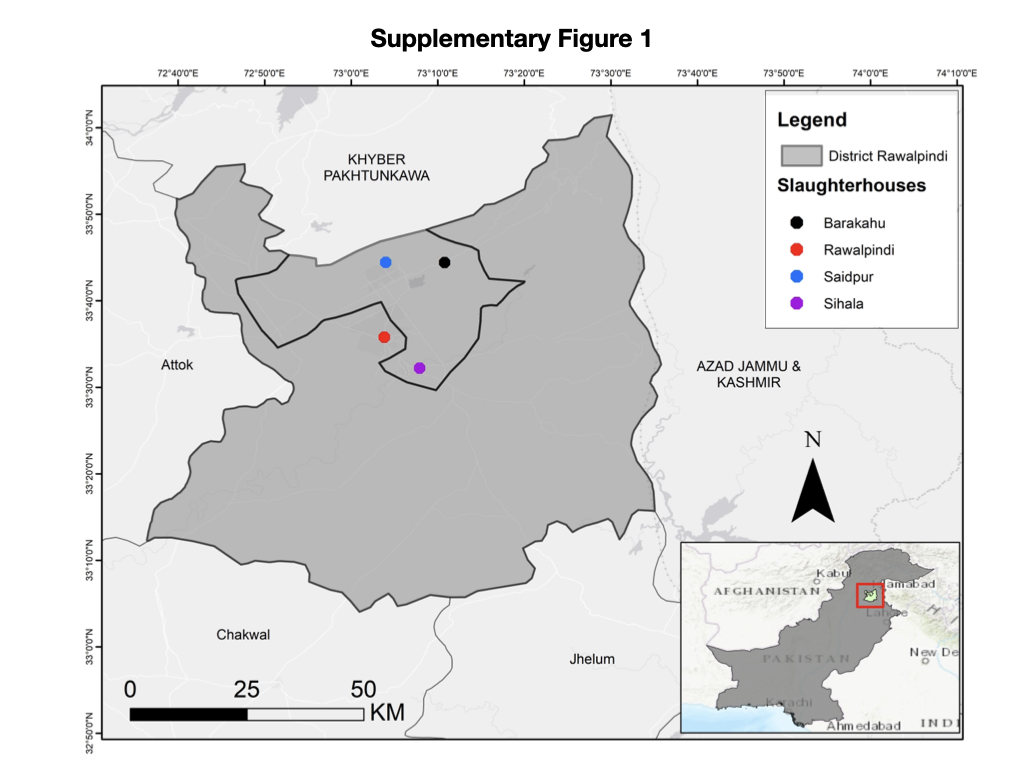

Supplement: Supplementary file 1 — Supplementary Material 1. Figure 1. Map showing the location of the slaughterhouses. [file 13071_2024_6626_MOESM1_ESM.tiff]

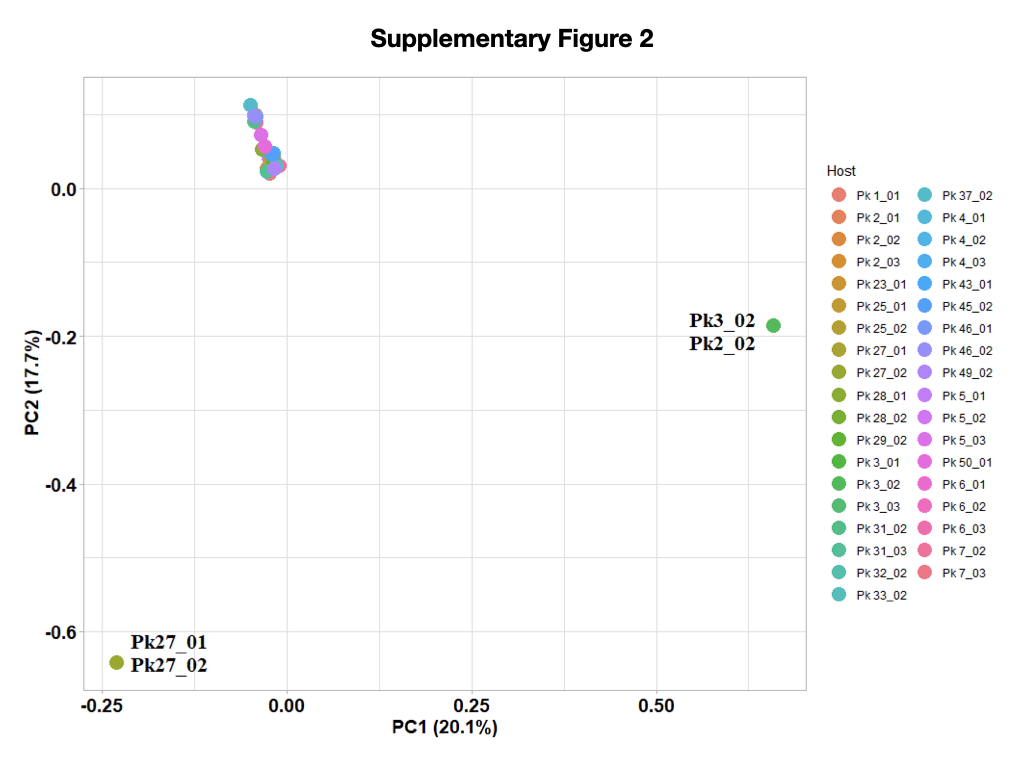

Supplement: Supplementary file 2 — Supplementary Material 2. Figure 2. Principal component analysis showing the first two principal components, which accounts for 38% of the variance. Larval sample are identified as host number_larva number, preceded by ‘pk’. These numbers correspond to those in Figure 1 where the preceding ‘pk’ is omitted. [file 13071_2024_6626_MOESM2_ESM.tiff]

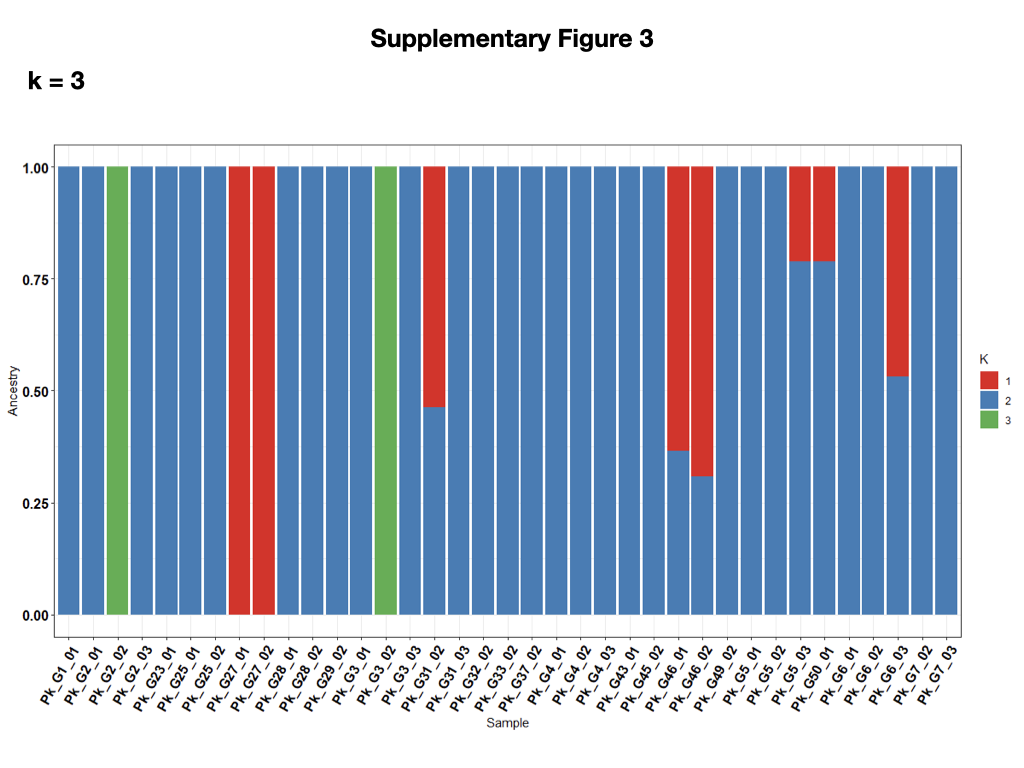

Supplement: Supplementary file 3 — Supplementary Material 3. Figure 3. ADMIXTURE analyses for k = 3, 7, and 15. [file 13071_2024_6626_MOESM3_ESM.zip › Supplementary Figure 3 001.tiff]

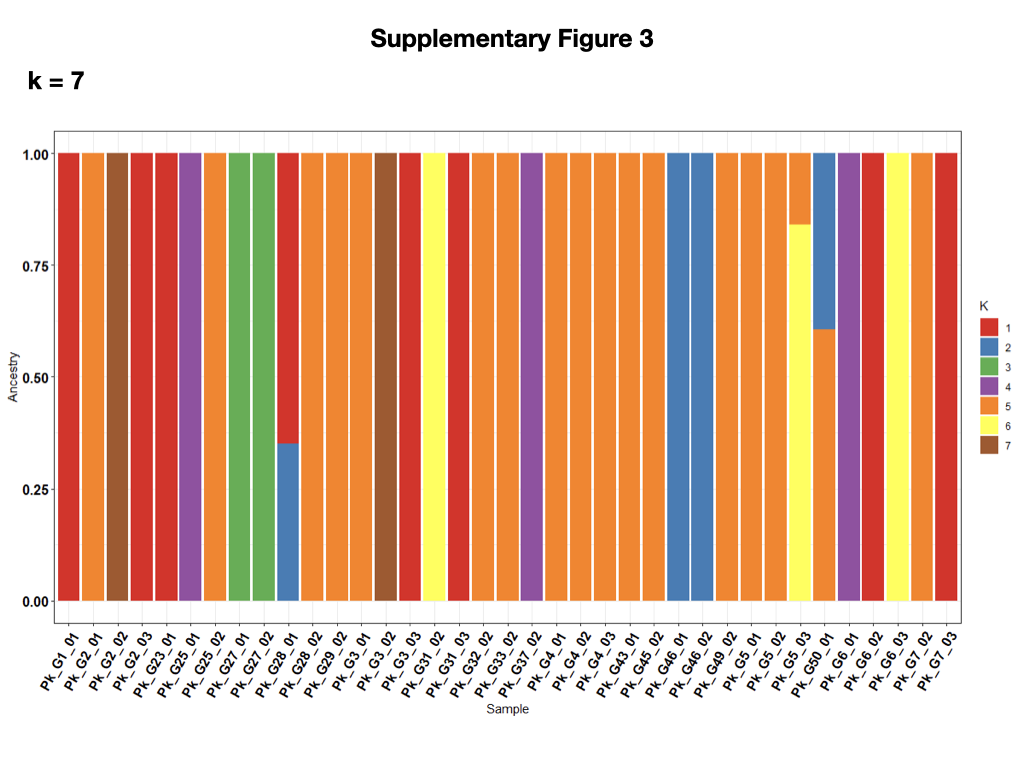

Supplement: Supplementary file 3 — Supplementary Material 3. Figure 3. ADMIXTURE analyses for k = 3, 7, and 15. [file 13071_2024_6626_MOESM3_ESM.zip › Supplementary Figure 3 002.tiff]

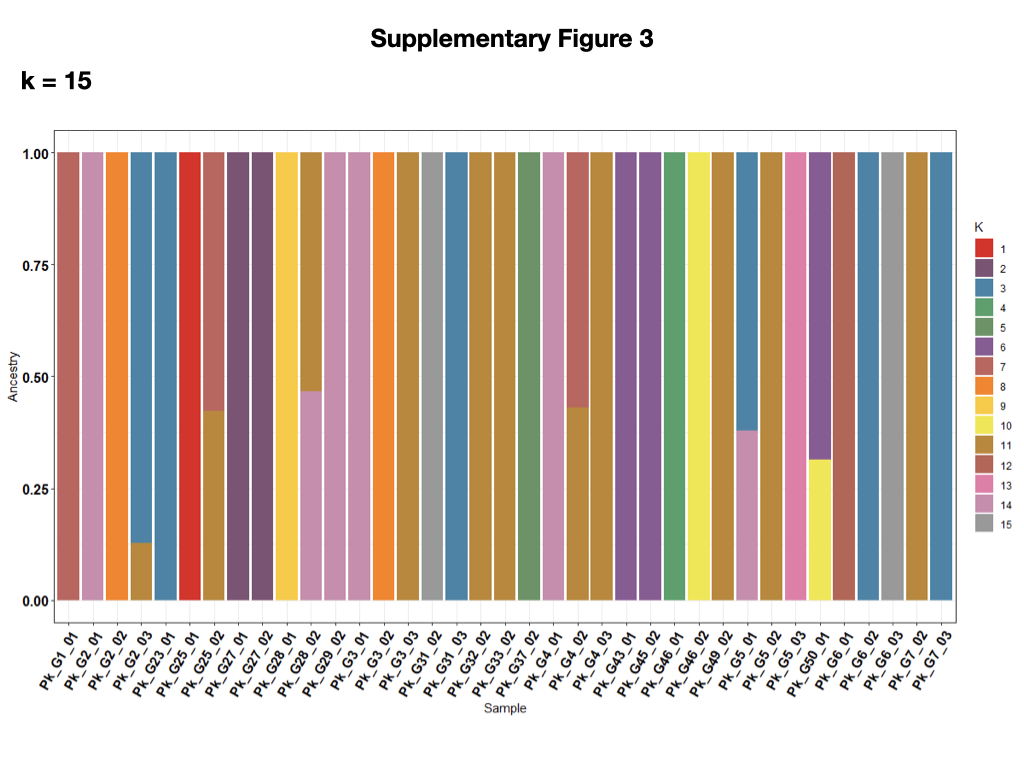

Supplement: Supplementary file 3 — Supplementary Material 3. Figure 3. ADMIXTURE analyses for k = 3, 7, and 15. [file 13071_2024_6626_MOESM3_ESM.zip › Supplementary Figure 3 003.tiff]
