# Supplementary material for: The genetic diversity of Strongyloides papillosus in Pakistani goats revealed by whole genome sequencing
Source: Parasit Vectors. 2024 Dec 20;17:527. doi: 10.1186/s13071-024-06626-6 (PMC11662772; doi:10.1186/s13071-024-06626-6)
Supplement: Supplementary file 4 — Supplementary Material 4. Table. [file 13071_2024_6626_MOESM4_ESM.docx]

**Supplementary Table 1.**

| **Sample Number** | **Age (years)** | **Collection date** | **No. iL3s harvested** | **No. libraries made** | **No. libraries passing QC** | **Sample ID** |
| --- | --- | --- | --- | --- | --- | --- |
| 1 | 0.6 | Aug 21 | 7 | 3 | 1 | pk1_01 |
| 2 | 1 | Aug 21 | 7 | 3 | 3 | pk2_01, pk2_02, pk2_03 |
| 3 | 1 | Oct 21 | 7 | 6 | 3 | pk3_01, pk3_02, pk3_03 |
| 4 | 1.4 | Nov 21 | 7 | 6 | 3 | pk4_01, pk4_02, pk4_03 |
| 5 | 1.1 | Dec 21 | 7 | 6 | 3 | pk5_01, pk5_02, pk5_03 |
| 6 | 1.3 | Feb 22 | 7 | 3 | 3 | pk6_01, pk6_02, pk6_03 |
| 7 | 1 | Feb 22 | 7 | 5 | 2 | pk7_02, pk7_03 |
| 8 | 3 | Feb 22 | 15 | 3 | 1 | pk23_01 |
| 9 | 2 | Feb 22 | 15 | 3 | 2 | pk25_01, pk25_02 |
| 10 | 2 | Feb 22 | 15 | 2 | 2 | pk27_01, pk27_02, |
| 11 | 4 | Feb 22 | 15 | 3 | 2 | pk28_01, pk28_02 |
| 12 | 4 | Feb 22 | 15 | 3 | 1 | pk29_02 |
| 13 | 3 | Feb 22 | 15 | 3 | 2 | pk31_02, pk31_03 |
| 14 | 3 | Feb 22 | 15 | 3 | 1 | pk32_02 |
| 15 | 3 | Feb 22 | 15 | 2 | 1 | pk33_02 |
| 16 | 3.5 | Mar 22 | 15 | 3 | 1 | pk37_02 |
| 17 | 2 | Apr 22 | 15 | 3 | 1 | pk43_01 |
| 18 | 2 | Apr 22 | 15 | 2 | 1 | pk45_02 |
| 19 | 2 | May 22 | 15 | 3 | 2 | pk46_01,pk46_02 |
| 20 | 2 | June 22 | 15 | 2 | 1 | pk49_02 |
| 21 | 2 | June 22 | 15 | 2 | 1 | pk50_01 |
|  | **2.1 (1.0)** |  | **259** | **69** | **37** |  |

QC = Quality Control. Sample ID is as Supplementary Figure 1 and Figure 1, though here the ‘pk’ is omitted for brevity; sample IDs in red are those showing evidence of admixture. The final row in bold shows the mean (SD) age and the total number of larvae and libraries.
